# Evaluation of Morphology and Location of Greater Palatine Foramen (GPF) in Different Sagittal Facial Types: A CBCT Study in an Iranian Adult Population

**DOI:** 10.1155/ijod/6654674

**Published:** 2025-12-22

**Authors:** Fatemeh Akbarizadeh, Shabnam Ajami, Kiarash Fereidouni, Mohsen Khaksari

**Affiliations:** ^1^ Orthodontic Research Center, School of Dentistry, Shiraz University of Medical Science, Shiraz, Iran, sums.ac.ir; ^2^ Student Research Committee, School of Dentistry, Shiraz University of Medical Sciences, Shiraz, Iran, sums.ac.ir

**Keywords:** CBCT, facial type, greater palatine foramen, maxilla

## Abstract

**Aim:**

The greater palatine foramen (GPF) transmits the greater palatine neurovascular bundle, and its position and shape directly affect palatal anesthesia, palatal surgery, and graft harvesting. This study aimed to evaluate variations in the location and morphology of the GPF across different sagittal maxillary growth patterns in an Iranian adult sample.

**Methods:**

Sixty cone beam computed tomography (CBCT) scans were analyzed (20 per Sella‐Nasion‐A (SNA) angle: deficient, normal, excessive). GPF shape, location relative to the molar teeth, its position with respect to adjacent structures (including distances to the alveolar ridge, mid‐palatal suture, contralateral GPF, and incisive foramen [IF]), and the angle between the mid‐palatal suture, IF midpoint, and GPF midpoint were assessed and compared among the three sagittal maxillary growth patterns.

**Results:**

The GPF‐IF distance differed significantly between sagittal groups (right: *p* = 0.004; left: *p* = 0.005), being shorter in the deficient maxillary group. The distribution of the GPF position relative to the molar teeth also differed significantly between groups (both sides: *p*  < 0.001). Other morphometric parameters showed no significant differences (Table 1).

**Conclusion:**

In this Iranian adult sample sagittal maxillary growth pattern is associated with variations in GPF‐IF distance and its anteroposterior position relative to molars; these should be considered during palatal anesthesia and surgical planning. Further clinical correlation is warranted.

## 1. Introduction

The hard palate is primarily composed of the palatine processes of the maxilla and the horizontal plates of the palatine bones [1]. There are several small foramina in the hard palate; the most clinically relevant are the greater palatine foramen (GPF) and the lesser palatine foramen [2]. The GPF, located posterolaterally on the hard palate, transmits the greater palatine nerve and vessels—structures of clear importance during palatal anesthesia, palatal surgeries, and graft harvesting [2–4]. Because the success and complication rates of palatal procedures (for example, nerve blocks and graft harvesting) depend on local anatomy, precise knowledge of GPF position reduces the risk of hemorrhage, nerve injury, and failed anesthesia [5–7].

Several studies have been carried out in the last decade to assess the morphology and location of GPF and the potential factors affecting these parameters. Three‐dimensional (3D) imaging techniques are prioritized over traditional two‐dimensional images due to their ability to avoid the drawbacks of superimposition and distortions found in 2D images. Conventional methods, such as periapical or panoramic radiographs and anatomical studies of dry skulls, have been used to examine the GPF; however, these approaches provide only limited or indirect information [8]. Multidetector computed tomography (MDCT) and magnetic resonance imaging (MRI) can also visualize craniofacial structures. Still, MDCT involves higher radiation exposure, while MRI offers lower spatial resolution for bony landmarks [9]. Currently, cone beam computed tomography (CBCT) is acknowledged as a valuable tool for clinicians to provide a 3D model of the maxillary bone due to its higher spatial resolution and lower dosage than MDCT [10]. CBCT enables precise determination of GPF position and morphology while minimizing radiation compared with MDCT [11, 12].

Aoun et al. [13] measured GPF diameter and position in a Lebanese CBCT sample and provided normative distances but noted limited sample size and single‐center bias. Rapado‐González et al. [14] described greater palatine canal (GPC) shape and path variants in an imaging cohort, highlighting anatomic variants relevant to nerve block technique but not correlating these variants with craniofacial growth patterns. Fonseka et al. [2] reported GPF positional measures in a Sri Lankan sample and emphasized interpopulation differences, but the cohort size limited subgroup analyses. More recent morphologic overviews [15] and systematic syntheses [16] consolidate these findings and underline that inconsistent landmark definitions and small or demographically narrow samples are major limitations across studies. Together, these works map GPF variability across populations but leave open the question of whether sagittal maxillary growth patterns systematically influence GPF anatomy.

The distance of GPF has been evaluated relative to the surrounding structures, including the midline maxillary suture, posterior border of the hard palate, and incisive foramen (IF) [16–18]. Many CBCT and dry‐skull investigations have reported population‐level variability in GPF shape and position [16, 18].

GPF cross‐sectional shape ‐ commonly classified as round or oval (with anterior–posterior or mediolateral elongation)—may influence needle placement and anesthetic spread during palatal blocks [19]. The most frequently reported shape is the oval type, subdivided as elongated in the anterior–posterior or mediolateral axis, with a pooled prevalence of 77.8% of specimens [16, 20]. Clinically, GPF shape and location influence anesthetic dispersion and the ease of identifying the foramen during procedures [15, 21]. An elongated foramen offers a larger target area for needle placement, which may help with localization and increase the chances of a successful palatal nerve block [15, 21].

Moreover, the anterior–posterior location of the GPF was evaluated in relation to the molar teeth across various populations. Most studies report the GPF located opposite the third molar in many populations [22], although some populations show different patterns—for example, placement between the second and third molars in an adult Chinese series [23]. These population‐level differences underline the need for population‐specific data [16].

Furthermore, a study evaluated the location and morphology of the GPF in different facial types in the vertical dimension. The findings of this study revealed that the GPF location regarding the palatal alveolar ridge (PAR), and relative to the molars, varies in different vertical facial types [19].

Recent CBCT and dry‐skull investigations have quantified GPF morphology across populations and highlighted methodological limitations that affect comparability. Although Kang et al. [4] provided 3D reference measures and described age‐related positional changes of the GPF, differences in GPF position and morphology across defined adult sagittal skeletal (maxillary) patterns have not been directly compared. Taken together, prior studies characterize population‐level GPF variability but do not directly address whether sagittal (Sella‐Nasion‐A [SNA]‐defined) maxillary growth patterns alter GPF morphometry; because sagittal growth (prognathism/retrognathism) changes the anteroposterior relationships of the midface and dentition, it may therefore alter the relative position of the GPF to molars and midline landmarks with direct implications for palatal anesthesia, surgical access, and graft planning. This study, therefore, evaluates GPF morphology and location across three sagittal growth patterns classified by the SNA angle in Iranian adults to fill this gap. We hypothesized that the sagittal maxillary growth pattern alters the anteroposterior relationship of the GPF to dental and midline landmarks.

## 2. Methods

### 2.1. Study Design and Ethics

This study was approved by the Research Ethics Committees of the School of Dentistry, Shiraz University of Medical Sciences (protocol number IR.SUMS.DENTAL.REC.1402.057). This cross‐sectional study is reported in accordance with the STROBE (Strengthening the Reporting of Observational Studies in Epidemiology) guidelines. A completed STROBE checklist is provided with the submission (Supporting Information File 1). Written informed consent for secondary use of anonymized data was obtained from all participants.

### 2.2. Sample Size Determination

Prior to data collection, an a priori sample size calculation using 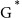
Power 3.1 based on pilot CBCT data (*n* = 5 per pilot group; means 36.54, 37.19, 37.67 mm for GPFIF) produced an effect size of *f* = 0.4631. For α = 0.05 and power = 0.80, the required total sample was ≈ 51. We collected *n* = 60 (20 per group) to increase accuracy and allow for exclusions.

### 2.3. Participants and Selection

From an archive of 300 CBCT scans acquired for diagnostic purposes (April 2019–December 2023), we consecutively screened and selected scans that met the inclusion/exclusion criteria until each SNA group quota (*n* = 20) was reached.

The inclusion criteria were high‐quality radiographs with a full‐face field of view of subjects aged >20 years. Vertical pattern of the subjects was also matched due to its potential effect on GPF characteristics. For this reason, we used the Björk analysis as a reliable indicator of vertical pattern. Since Lacerda‐Santos et al. [19] reported that the location of GPF varies among different facial types, only subjects with a Björk sum of 390°–400° (normal vertical pattern) were included to control vertical growth as a confounder.

The exclusion criteria were patients with a previous history of trauma, surgery, pathologies in the maxilla, cleft lip/palate subjects, and edentulous arches in the molar area. Images with extensive restorations causing misleading artifacts were also excluded from the study.

### 2.4. Imaging Protocol

All scans were obtained at a single radiology center using a VGI evo NewTom ENT CBCT system (QR S.R.L., Verona, Italy) with 0.3 mm voxel size, 110 kVp, and 7.56 mAs. Measurements were taken using NNT Viewer software (NNT 9.21, Image Works, Verona, Italy). Scans were acquired using the center’s standard stabilization protocol (natural head position with cross‐light alignment and head‐holder stabilization) to minimize tilt and motion.

### 2.5. Sagittal Classification (SNA and Cranial‐Base Adjustment)

For equal distribution of sagittal maxillary growth patterns, we categorized the subjects into three groups using the SNA angle. The SNA angle was chosen because it would not change considerably with age. We adjusted SNA for cranial‐base angulation by calculating SNA′ = SNA + (SN‐FH ‐ 7°); participants were classified as deficient (SNA′ < 80°), normal (SNA′ = 80–84°), or excessive (SNA′ > 84°) [24]. We used 7° as the reference SN‐FH value (midpoint of normal range 6°–9°).

### 2.6. Measurements

We then assessed the shape of GPF, its position relative to adjacent structures, and its proximity to the molar teeth, and compared these between the three sagittal maxillary growth patterns.

GPF shape was measured as the ratio of anteroposterior length to mediolateral width: ratio < 0.95 = mediolaterally elongated; 0.95–1.05 = round; > 1.05 = anteroposteriorly elongated [25] (Figure 1A,B).

Figure 1The morphology of the GPF. The mediolateral width of the GPF (A), and the anterior–posterior length (B).

**Figure:**
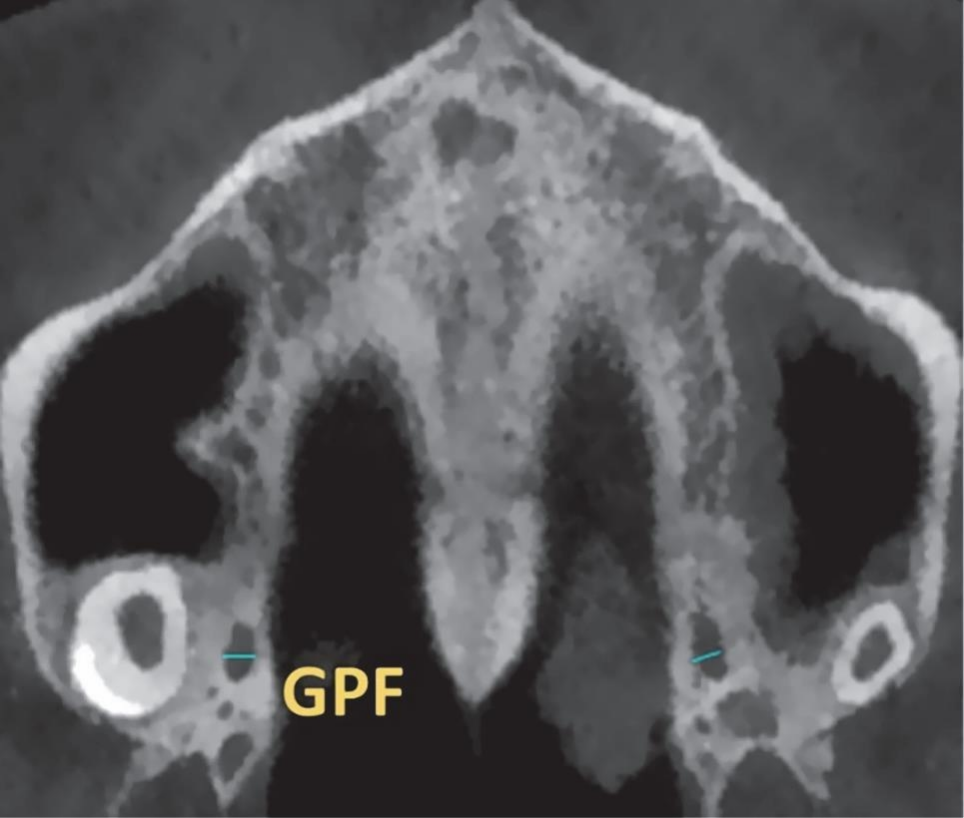
(A)

**Figure:**
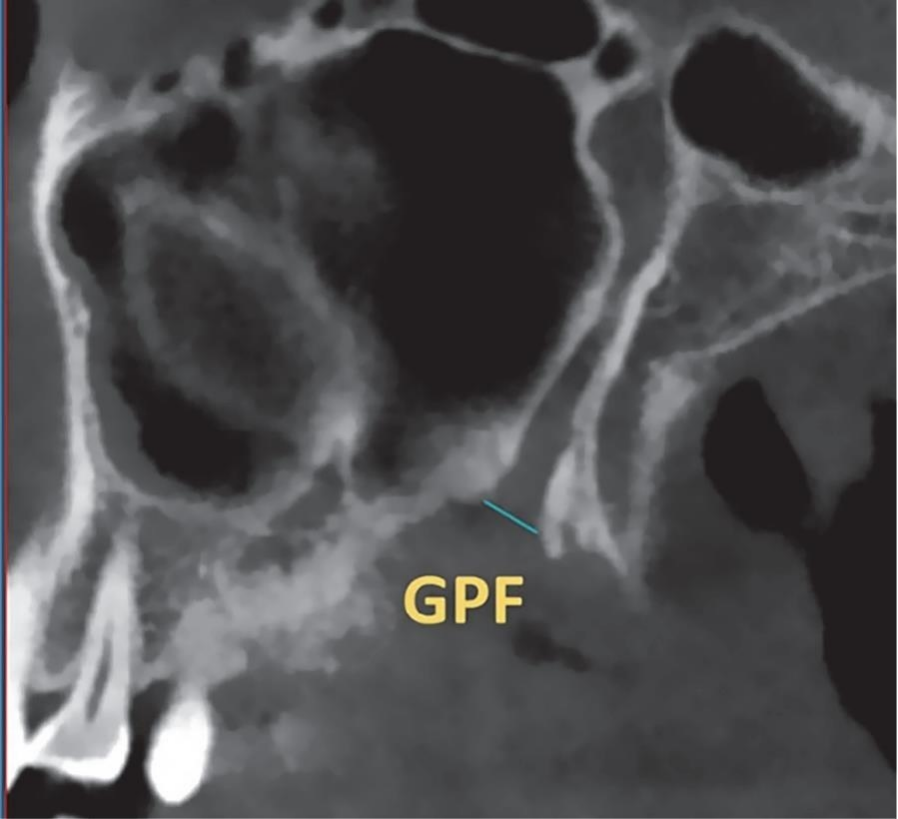
(B)

The position of GPF relative to the surrounding structures was measured as follows: The distances between GPF and the most coronal point of the PAR, the mid‐palatal suture, the other GPF, and the midpoint of the IF. Also, the angle formed between the mid‐palatal suture, the IF’s midpoint, and the GPF’s midpoint was measured on both sides (Figure 2). Except for the PAR distance, which was done in coronal cut, all the other measurements were carried out in axial cut.

Figure 2Linear and angular measurements of GPF relative to the surrounding structures. Mid‐palatal suture (A), the opposing side GPF (B), midpoint of incisive foramen (C), the angle formed between GPF‐IF‐PS (D), distance between GPF, and the most coronal point of the alveolar ridge (E).

**Figure:**
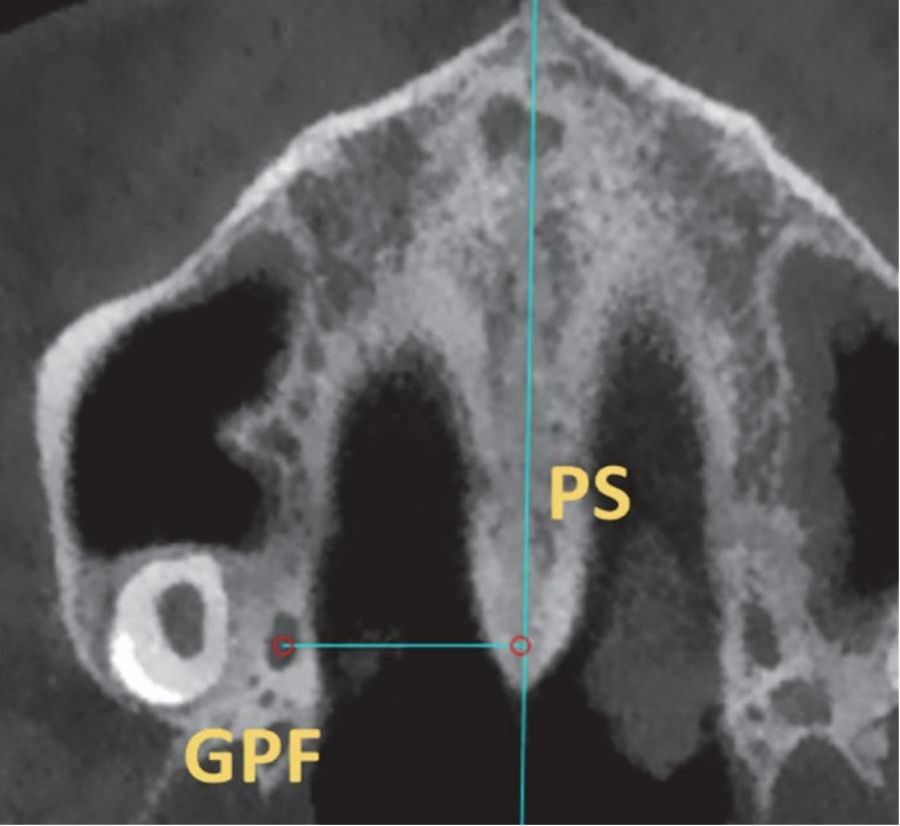
(A)

**Figure:**
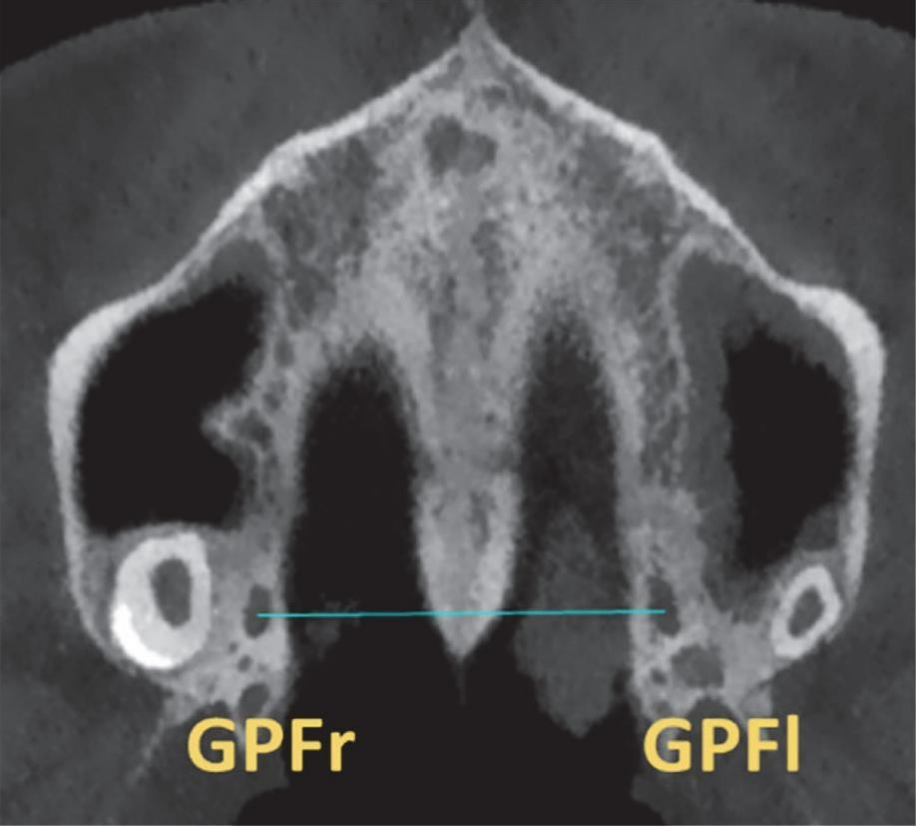
(B)

**Figure:**
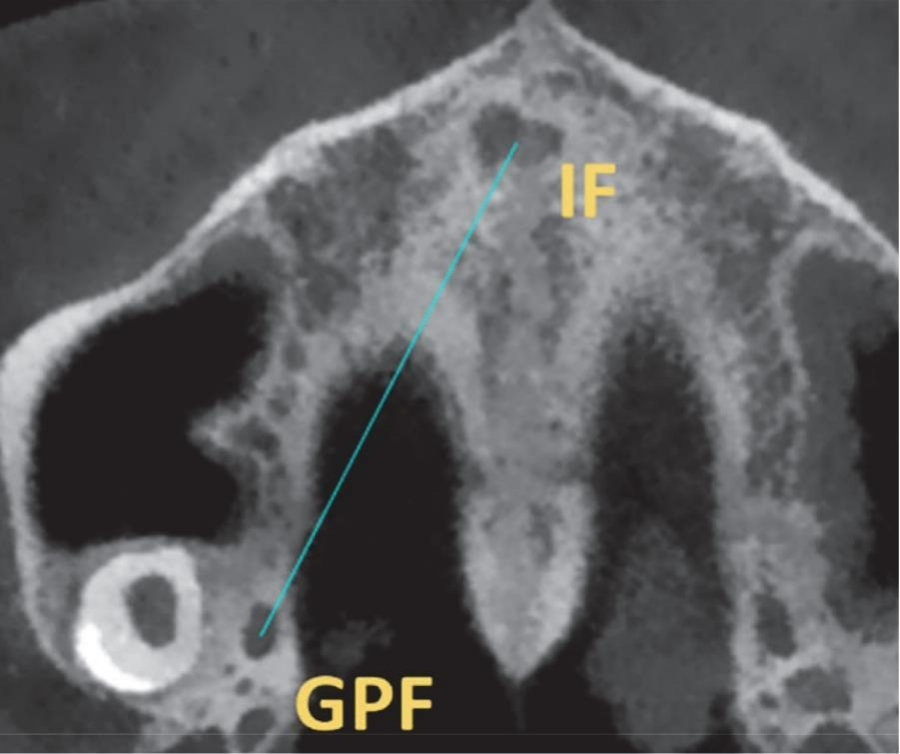
(C)

**Figure:**
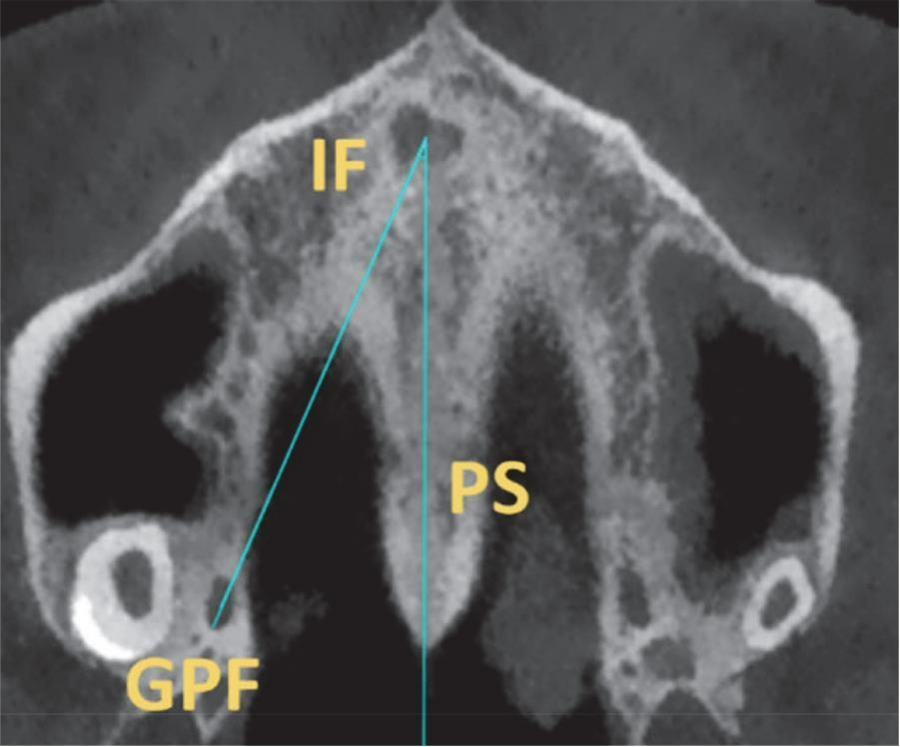
(D)

**Figure:**
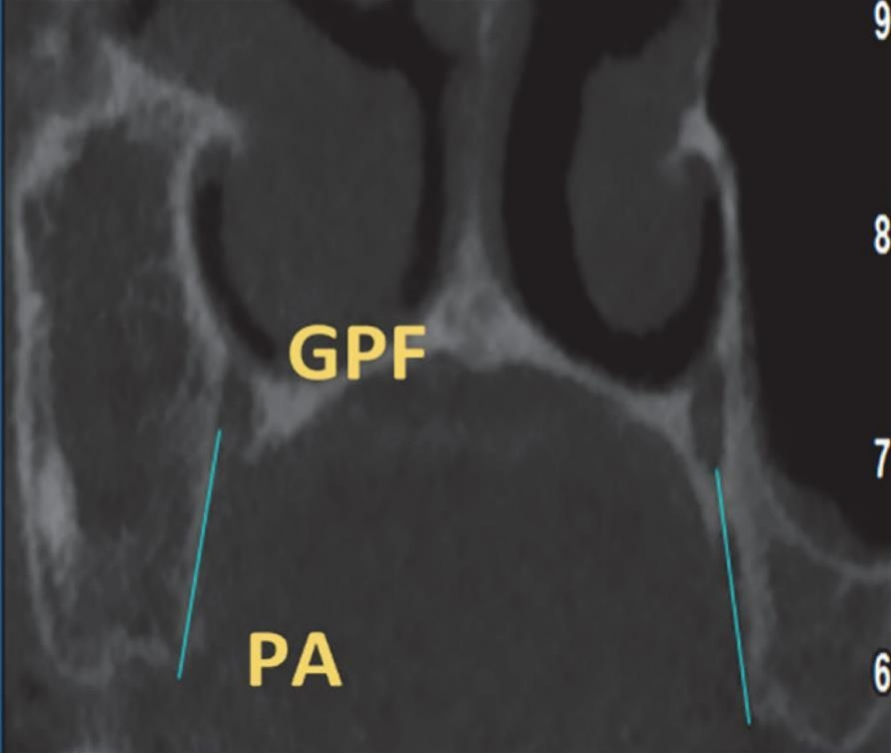
(E)

**Figure 3:**
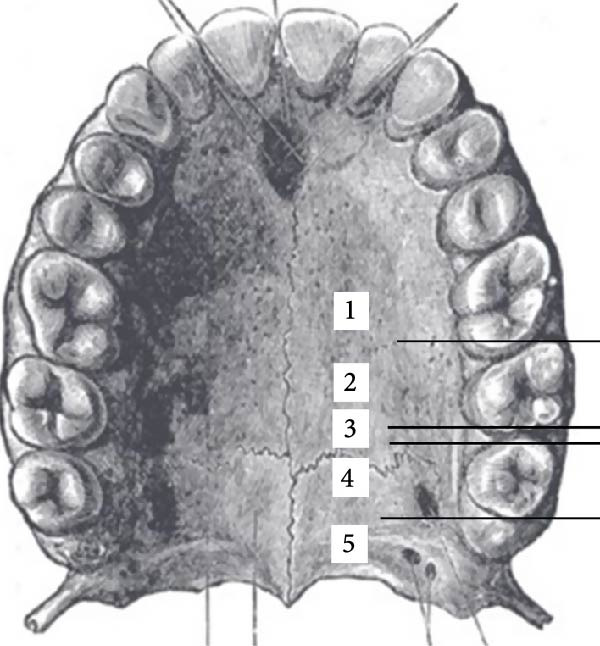
Position types of GPF regarding the molar teeth.

The anterior–posterior location of the midpoint of the GPF relative to the molar teeth was assessed in axial dimension and categorized into five groups: Type 1: GPF mesial to the second molar; Type 2: opposite the second molar; Type 3: between the second and third molars; Type 4: opposite the third molar; Type 5: distal to the third molar (Figure 3).

### 2.7. Examiner Training, Blinding, and Calibration

A final‐semester dentistry student (trained for this project) and an oral and maxillofacial radiologist (assistant professor with >5 years of CBCT experience) performed measurements independently. Raters were blinded to subject SNA classification and to each other’s results during measurement sessions. Calibration comprised the student analyzing 200 separate CBCTs (not included in the study) under the radiologist’s supervision, followed by assessment on 10 pilot CBCTs; calibration was deemed successful when student‐radiologist agreement exceeded intraclass correlation coefficient (ICC) = 0.90 on the pilot set.

### 2.8. Reliability Analysis

Each rater completed the full measurement set independently in two sessions (each lasting 120 min) with a 3‐day interval between sessions. To assess intraobserver reliability, the radiologist repeated measurements on 15 randomly selected scans (25% of the sample) after a 2‐week interval. The required sample size for reliability testing (α = 0.05, power = 80%, expected ICC = 0.90) was *n* = 13; therefore, 15 scans were included. Inter‐ and intraobserver reliability were calculated using a two‐way random‐effects model for single measures and absolute agreement.

### 2.9. Statistical Analysis

All measurements taken were entered into SPSS (Statistical Package for Social Sciences for Windows, Inc., USA) version 26. The paired *t*‐test, and one‐way ANOVA were used to assess morphometric linear and angular data. Wilcoxon signed rank test and Chi‐square were also used to determine the relation between maxillary growth patterns and the location of the foramen relative to the molars. The confidence interval (CI) of 95% and the *p*  < 0.05 were considered the statistical significance level. The intra‐ and interobserver ICC coefficients were used to assess the reliability of the study.

## 3. Results

A total of 60 subjects were analyzed (28 male, 32 female; mean age 33.78 years, range 20–60). There were no statistically significant differences in GPF morphology between the three sagittal groups (*p*  > 0.05). GPF morphology was anteriorposteriorly elongated in 59 participants; one participant had a round foramen. Among morphometric measures, only the GPF‐IF distance differed significantly between groups (right *p* = 0.004; left *p* = 0.005; Table 1). Post‐ hoc comparison between the GPF‐IF distance individually between the three groups reported that this measurement was statistically lower in the deficient maxillary growth pattern group in comparison with the two other groups (*p*  < 0.05; Table 2).

**Table 1:** Comparison of linear and angular parameters between different sagittal maxillary growth patterns (mean ± SD).

Variable	Side/type	Deficient (n = 20) mean ± SD	Normal (n = 20) mean ± SD	Overgrowth (n = 20) mean ± SD	*p*‐Value
GPF‐PS (mm)	Right	15.09 ± 1.24	15.54 ± 1.21	15.76 ± 1.62	0.298
GPF‐PS (mm)	Left	14.93 ± 1.34	15.33 ± 1.76	15.88 ± 1.60	0.169
INTERFORAMEN (mm)	Bilateral	30.37 ± 2.35	31.64 ± 3.46	32.03 ± 3.10	0.196
GPF‐IF (mm)	Right	35.04 ± 3.18	37.19 ± 2.64	38.17 ± 2.95	**0.004** ^∗^
GPF‐IF (mm)	Left	35.65 ± 3.17	38.14 ± 2.18	38.45 ± 2.83	**0.005** ^∗^
GPF‐IF‐PS angle (°)	Right	25.42 ± 2.27	23.79 ± 2.02	24.50 ± 2.87	0.112
GPF‐IF‐PS angle (°)	Left	24.41 ± 2.98	23.21 ± 3.38	24.35 ± 2.80	0.353
GPF‐PAR (mm)	Right	12.25 ± 2.74	12.17 ± 1.63	11.68 ± 2.31	0.245
GPF‐PAR (mm)	Left	12.27 ± 2.67	12.22 ± 1.74	11.71 ± 2.11	0.716

*Note*: *p*‐Values from one‐way ANOVA; *n* = 20 per group. The bold indicates *p*‐values lower than 0.05, which are statistically significant.

Abbreviations: GPF, greater palatine foramen; IF, incisive foramen; PAR, palatine alveolar ridge; PS, palatine suture; SD, standard deviation.

^∗^ANOVA one‐way *p*‐value < 0.05; LSD post hoc (*p*‐value < 0.05).

**Table 2:** Pairwise comparisons of GPF‐IF distance between sagittal maxillary growth patterns (LSD test).

Comparison (group 2 ‐ group 1)	Mean difference (mm)	95% CI for difference (mm)	*p*‐Value	Cohen’s *d*
Right GPF‐IF (mm)				
Normal‐deficient	2.15	0.28 to 4.02	0.024 ^∗^	0.74
Overgrowth‐deficient	3.13	1.17 to 5.09	0.001 ^∗^	1.02
Overgrowth‐normal	0.98	−0.81 to 2.77	0.296	0.35
Left GPF‐IF (mm)				
Normal‐deficient	2.49	0.75 to 4.23	0.006 ^∗^	0.92
Overgrowth‐deficient	2.80	0.88 to 4.72	0.002 ^∗^	0.93
Overgrowth‐normal	0.31	−1.31 to 1.93	0.724	0.12

Abbreviations: GPF, greater palatine foramen; IF, incisive foramen.

^∗^Significant results.

There was a statistically significant difference between the three studied groups on both sides in the frequency of GPF position relative to the molar teeth (Table 3; *p*  < 0.001 for both sides). The predominant location of GPF relative to the molar tooth was mesial to the third molar tooth in the deficient maxillary growth type, opposite to the third molar tooth in the normal maxillary growth type, and distal to the third molar tooth in the growth excess maxillary type.

**Table 3:** GPF position relative to the molars in different sagittal facial types.

Right GPF molar type						*p*‐Value
	1	2	3	4	5	
Deficient	0.0%	40.0%^a^	20.0%	40.0%	0.0%	<0.001 ^∗^
Normal	0.0%	0.0%	10%	75%^a^	15%	
Excessive	0.0%	0.0%	0.0%	45.0%	55.0%^a^	

**Left GPF molar type**						***p*-Value**
	**1**	**2**	**3**	**4**	**5**	

Deficient	0.0%	25.0%^a^	30.0%^a^	45.0%	0.0%	<0.001 ^∗^
Normal	0.0%	0.0%	05%	85%^a^	10%	
Excessive	0.0%	0.0%	0.0%	40.0%	60.0%^a^	

Abbreviation: GPF, greater palatine foramen.

^∗^ *p*‐Value from chi‐square test.

^a^The most prevalent type.

Except for the angle formed between GPF‐IF‐PS, the other linear and angular measurements were significantly different between the two genders (Table 4).

**Table 4:** Comparison of linear and angular measurements between males and females.

Variable	Sex	Mean ± SD	95% CI for mean	*p*‐Value	Cohen’s *d* (male–female)
GPF‐PS (mm)	Male	15.78 ± 1.45	15.22–16.34	0.014 ^∗^	0.46
	Female	15.11 ± 1.46	14.58–15.64		

INTERFORAMEN (mm)	Male	32.24 ± 3.24	30.98–33.50	0.033 ^∗^	0.57
	Female	30.57 ± 2.67	29.60–31.54		

GPF‐IF (mm)	Male	38.00 ± 2.81	36.91–39.09	0.002 ^∗^	0.56
	Female	36.32 ± 3.13	35.19–37.45		

GPF‐IF‐PS angle (°)	Male	23.83 ± 2.59	22.83–24.83	0.086	‐0.31
	Female	24.68 ± 2.79	23.67–25.69		

GPF‐PAR (mm)	Male	12.84 ± 1.85	12.12–13.56	<0.001 ^∗^	0.70
	Female	11.37 ± 2.28	10.54–12.20		

*Note*: Means ± SD, 95% CI for the mean, and *p*‐values from independent t‐tests. Cohen’s *d* is calculated as male–female.

Abbreviations: GPF, greater palatine foramen; IF, incisive foramen; PAR, palatine alveolar ridge; PS, palatine suture; SD, standard deviation.

^∗^Significant results.

As shown in Table 5, comparing linear and angular measurements between the two sides revealed no significant difference except in the GPF‐IF distance.

**Table 5:** Comparison of linear and angular measurements between right and left sides.

Variable	Side	Mean ± SD	95% CI for mean	*p*‐Value	Cohen’s *d* (right–left)
GPF‐PS (mm)	Right	15.47 ± 1.38	15.11–15.83	0.536	0.06
	Left	15.38 ± 1.60	14.97–15.79		

GPF‐IF (mm)	Right	36.80 ± 3.17	35.98–37.62	<0.001 ^∗^	−0.20
	Left	37.41 ± 2.99	36.64–38.18		

GPF‐IF‐PS angle (°)	Right	24.57 ± 2.47	23.93–25.21	0.066	0.21
	Left	23.99 ± 2.94	23.23–24.75		

GPF‐PAR (mm)	Right	12.03 ± 2.25	11.45–12.61	0.145	−0.01
	Left	12.06 ± 2.18	11.50–12.62		

*Note*: Means ± SD, 95% CI for the mean, and *p*‐values frompaired t‐tests. Cohen’s *d* is calculated as right–left (unpaired approximation).

Abbreviations: GPF, greater palatine foramen; IF, incisive foramen; PAR, palatine alveolar ridge; PS, palatine suture; SD, standard deviation.

^∗^Significant results.

Interobserver variability was evaluated using the ICC based on a two‐way random effects model (ICC = 0.939). To assess intraobserver reliability, the radiologist repeated all measurements on 15 randomly selected images after a 2‐week interval. The ICC value for intraobserver reliability was 0.987, indicating excellent consistency.

## 4. Discussion

Insufficient information regarding the GPF location during surgical procedures may cause complications such as severe blood loss, necrosis, and greater palatine nerve impairment [26]. As a result, precise localization of the GPF using multiple anatomical reference points is essential to avoid potential injuries to this region. Different growth patterns have diversity regarding their craniofacial growth, muscular activity, and occlusion [27, 28]. Due to these diversities, the location of the GPF in different sagittal growth patterns is assessed and compared with each other in the present study.

### 4.1. Comparison With Previous Literature

The distances between the GPF‐PS and GPF‐IF measured in the present study are important factors for the assessment of the width and length of the graft for the restoration of the bony palate after a traumatic injury or surgery [3]. Our GPF‐PS distances were consistent with prior CBCT studies [11, 15, 21, 29–31]. GPF‐IF distances fell within published ranges [11, 15, 21, 29–31]. These comparator cohorts represent diverse populations (e.g., Middle Eastern, South Asian, East Asian, and North African), underscoring interpopulation variability. Inter‐foramen distances were similar to Sheikhi et al. [32]. The other distance from the anatomical landmark was the GPF‐PAR distance, which is especially important in edentulous ridges for locating the GPF. Our GPF‐PAR distances were larger than Lacerda‐Santos et al. [19] and Ikuta et al. [11], but close to Zaghden et al. [33], possibly reflecting ethnic/sample differences. Such differences may also reflect methodological factors (landmark definitions, voxel size, field of view, dental status). The GPF‐IF‐PS angle was comparable to published values [16, 34, 35]. Accordingly, our values should be interpreted as population‐specific reference data for Iranian adults rather than generalized to other populations.

Most prior work that stratified by vertical facial type [19] reported differences primarily in GPF‐PAR and related measures of palatal depth/ridge proximity, with modest effects on anteroposterior relationships. In contrast, our sagittal stratification (SNA‐defined) showed group differences in anteroposterior metrics—specifically GPF‐IF distance and molar‐position type—while GPF shape and most other distances did not differ among groups. This pattern suggests that vertical variation mainly influences superoinferior palatal form (how close the foramen lies to the PAR), whereas sagittal variation primarily alters anteroposterior relationships between the GPF, IF, and molars. Accordingly, the deficient group showed shorter GPF‐IF and more mesial GPF‐molar relationships, while the excessive group showed longer GPF‐IF and more distal relationships ‐ consistent with anteroposterior displacement of the maxilla relative to the cranial base. Apparent discrepancies in the literature (e.g., reports of anterior GPF position in Class II samples) [36] likely reflect uncontrolled vertical pattern, as noted by Lacerda‐Santos et al. [19]; our design held vertical pattern constant (Björk‐normal), isolating sagittal effects.

Regarding the morphology of the GPF, except for one subject who had a round morphology, all the other participants revealed elongation in the anterior–posterior morphology, in accordance with the findings of Lacerda‐Santos et al. [19] and Kim et al. [16]. This elongated morphology can be attributed to the eruption of the teeth, which increases the length of the palate.

We used the molar‐type mapping approach which was introduced by Tomaszewska et al. [22]. They presented a classification considering three tangents drawn parallel to the interproximal area of the upper molars, dividing the area into five sections. In the current study, the position of the GPF regarding the molar teeth was reported to be opposite to that of the third molar tooth in most participants. This finding is in accordance with several studies on this issue [11, 16, 18, 34, 37–39].

Comparing the position of GPF relative to the molar teeth between the three sagittal growth patterns, we found that the predominant position was only opposite to the third molar tooth in the normal maxillary growth type. While in the deficient and growth excess maxillary growth patterns, the predominant location was mesial to the third molar and distal to the third molar teeth, respectively. The explanation for this matter is that the growth of the jaw and development of the cranial structures do not necessarily correspond with each other [40, 41]. During growth, the cranium‐related structures, such as GPF, alter to a different degree from the jaw‐related structures, such as dentition [41]. The maxillary growth results in the anterior movement of both GPF and dentition [36], but due to the unequal movement rates between the teeth and the GPF, it has been observed that with normal maxillary growth, the GPF moves posteriorly relative to the molar teeth [42]. Any disturbance in this phenomenon due to the overgrowth or deficient maxillary growth results in an altered GPF position relative to the molars than normal. Lacerda‐Santos et al. [19], obtained similar results and reported significant differences between different vertical facial types. However, a study carried out by Rachana et al. [36], who compared the GPF location relative to the molar teeth between the three facial types and also a group of cleft subjects, revealed that in the class 2 subjects, the GPF was located more anteriorly than in the other groups. The justification for this disparity is their methodological limitation. Based on the study by Lacerda‐Santos et al. [19], the vertical growth pattern influences the GPF characteristics, necessitating the selection of subjects with similar vertical growth patterns. However, in the study conducted by Rachana et al. [36], the effect of vertical growth was overlooked. Our design held the vertical pattern constant, isolating sagittal effects, which likely explains the differing directionality.

We also compared the morphologic and morphometric parameters between the different sagittal growth patterns and found that the GPF‐IF mean distance and position related to the molar tooth varied significantly in different growth patterns. The growth excess group (forward position of the maxilla) expressed the highest GPF‐IF mean and the most posterior positioning relative to the molar teeth out of the three studied groups. Due to the lack of studies on this issue, we cannot compare this result with other articles. The sole study that compared these measurements between the three vertical facial types was conducted by Lacerda‐Santos et al. [19], who reported that one measurement was different between the types, which was the GPF‐PAR distance. We can justify our study’s findings by explaining the maxilla’s growth pattern. It was previously shown that the growth of the maxilla begins with an anterior–posterior lengthening of the jaw. Therefore, maxillary growth excess will lead to maxillary prognathism and an increased anterior–posterior length, which causes increased GPF‐IF distance [43–45].

Comparing the measured parameters between the two genders, we found that the linear measurements were significantly higher in males than in females. The justification for this result is that the cranial structures in males generally exhibit greater size than in females [46]. Sexual dimorphism of the cranium has been proven in the literature [22]. One major limitation in previous studies regarding GPF being carried out in skulls is the inability to determine the gender in skull studies, which makes such studies incomparable to the current article. Out of radiographic studies, other than the study done by Ikuta et al. [11], who reported no significant difference between the two genders, the results of the other papers were in line with the present study [19, 22, 30, 46].

The result of this study reveals that, except for the GPF‐IF, the other measured parameters do not show significant differences between the two sides. This finding is in accordance with the studies conducted by Zaghden et al. [33], Kalmin et al. [47], Lacerda‐Santos et al. [19], Sheikhi et al. [32], and Rachana et al. [36]. However, side‐related discrepancies have been reported in the Tomaszewaska et al. [8] study, which can be attributed to ethnical differences.

### 4.2. Clinical Significance

Our results demonstrate that in this Iranian sample, sagittal maxillary growth correlates with changes in the anterior–posterior relationships of the GPF, particularly the GPF‐IF distance and its position relative to the molars. Clinically, sagittal type may guide the site/angle for greater palatine nerve blocks and the dimensions/safety of palatal grafts. For example, an anteriorly positioned GPF in maxillary deficiency could change the optimal injection site/angle for a greater palatine nerve block and may limit the safe dimensions of palatal grafts, potentially affecting anesthesia success, bleeding risk, and graft viability. Systematic reviews report notable postoperative pain and bleeding after palatal grafting; tailoring anesthesia and graft planning to sagittal type may help reduce morbidity [48]. Prospective clinical studies should test whether sagittal‐tailored protocols improve anesthesia success and graft outcomes [49].

### 4.3. Study Limitations and Future Directions

Our study is a single‐center, cross‐sectional CBCT analysis of scans obtained for other diagnostic purposes, likely representing a single regional/ethnic population; therefore, its generalizability is limited. We did not perform multivariable adjusted analyses because the sample size limited the degrees of freedom. The group size (*n* = 20) was based on the pilot study (*n* = 15; 5 per pilot group) and the a priori power calculation described above. Nonsignificant results in this study should not be interpreted as proof of statistical equivalence, since no formal equivalence testing was performed. Instead, they indicate that large differences between groups were not observed, given the current sample size, and the 95% CIs show the range within which such differences could plausibly fall, consistent with our data. Future multicenter studies should include age, gender, and ethnicity as covariates. Future work should integrate CBCT anatomy with procedural endpoints (e.g., block success and hemostasis) to establish sagittal‐type decision aids.

## 5. Conclusion

This CBCT study found that sagittal maxillary growth relates to differences in GPF‐IF distance and the GPF’s anterior–posterior relation to molars. These differences could affect palatal anesthesia and surgery, but clinical studies are needed to confirm their functional significance.

## Ethics Statement

The protocol for the current study was approved by the Ethics Committee of Shiraz University of Medical Sciences. (Protocol number IR.SUMS.DENTAL.REC.1402.057).

## Conflicts of Interest

The authors declare no conflicts of interest.

## Author Contributions

Conceptualization, writing – review and editing: All authors. Methodology, investigation (data collection), formal analysis, data curation,: Fatemeh Akbarizadeh and Mohsen Khaksari. Writing – original Draft: Fatemeh Akbarizadeh and Shabnam Ajami.

## Funding

The authors would like to thank the Vice Chancellor for Research, Shiraz University of Medical Science, for supporting this investigation.

## Supporting Information

Additional supporting information can be found online in the Supporting Information section.

## Supporting information


**Supporting Information** Supporting File 1. STROBE checklist for cross‐sectional observational studies (STROBE statement).

## Data Availability

The data that support the findings of this study are available from the corresponding author upon reasonable request.
