# Supplementary material for: Evaluation of Morphology and Location of Greater Palatine Foramen (GPF) in Different Sagittal Facial Types: A CBCT Study in an Iranian Adult Population
Source: Int J Dent. 2025 Dec 22;2025:6654674. doi: 10.1155/ijod/6654674 (PMC12752903; doi:10.1155/ijod/6654674)
Supplement: Supplementary file 1 — Supporting Information Supporting File 1. STROBE checklist for cross‐sectional observational studies (STROBE statement). [file IJOD-2025-6654674-s001.docx]

STROBE Statement—Checklist of items that should be included in reports of ***cross-sectional studies***

|  | Item No | Recommendation | Page No |
| --- | --- | --- | --- |
| **Title and abstract** | 1 | (*a*) Indicate the study’s design with a commonly used term in the title or the abstract | **Title / Running title / Abstract** |
|  |  | (*b*) Provide in the abstract an informative and balanced summary of what was done and what was found | **Abstract** |
| Introduction | | | |
| Background/rationale | 2 | Explain the scientific background and rationale for the investigation being reported | **Introduction -** first 2–3 paragraphs (lines describing clinical importance and prior studies). |
| Objectives | 3 | State specific objectives, including any prespecified hypotheses | **Introduction** - last paragraph: “We hypothesized that the sagittal maxillary growth pattern alters the anteroposterior relationship...” |
| Methods | | | |
| Study design | 4 | Present key elements of study design early in the paper | **Methods - first paragraph**: “This study was approved... This cross-sectional study is reported in accordance with the STROBE...” |
| Setting | 5 | Describe the setting, locations, and relevant dates, including periods of recruitment, exposure, follow-up, and data collection | **Methods - Setting**: “From an archive of 300 CBCT scans acquired for diagnostic purposes (April 2019–December 2023) … single radiology center.” |
| Participants | 6 | (*a*) Give the eligibility criteria, and the sources and methods of selection of participants | **Methods - Participants/Inclusion-Exclusion**: age >20, Björk sum 390°–400°, exclusion criteria listed. |
| Variables | 7 | Clearly define all outcomes, exposures, predictors, potential confounders, and effect modifiers. Give diagnostic criteria, if applicable | **Methods - Variables**: GPF shape ratio, SNA′ classification, distances (GPF–IF, GPF–PS, GPF–PAR), angle GPF–IF–PS; vertical pattern controlled. |
| Data sources/ measurement | 8* | For each variable of interest, give sources of data and details of methods of assessment (measurement). Describe comparability of assessment methods if there is more than one group | **Methods - Imaging & measurement**: VGI evo NewTom ENT CBCT (0.3 mm voxel), NNT Viewer 9.21; measurement planes described (axial/coronal). |
| Bias | 9 | Describe any efforts to address potential sources of bias | **Methods — Rater blinding & calibration**: blinded raters, calibration protocol (student analyzed 200 CBCTs under supervision; pilot ICC >0.90). |
| Study size | 10 | Explain how the study size was arrived at | **Methods - Sample size**: a priori G*Power (effect size f = 0.4631), required ≈51, n collected = 60 (post-hoc power 0.89). |
| Quantitative variables | 11 | Explain how quantitative variables were handled in the analyses. If applicable, describe which groupings were chosen and why | **Methods - Quantitative handling**: GPF shape ratio cutoffs (<0.95, 0.95–1.05, >1.05); SNA′ thresholds (<80°, 80–84°, >84°). |
| Statistical methods | 12 | (*a*) Describe all statistical methods, including those used to control for confounding | **Methods - Statistical Analysis**: paired t-test, independent t-test, one-way ANOVA, Wilcoxon, Chi-square, ICC. |
|  |  | (*b*) Describe any methods used to examine subgroups and interactions |  |
|  |  | (*c*) Explain how missing data were addressed |  |
|  |  | (*d*) If applicable, describe analytical methods taking account of sampling strategy |  |
|  |  | (*e*) Describe any sensitivity analyses |  |
| Results | | | |
| Participants | 13* | (a) Report numbers of individuals at each stage of study—eg numbers potentially eligible, examined for eligibility, confirmed eligible, included in the study, completing follow-up, and analysed | **Methods/Results**: “From an archive of 300 CBCT scans… screened and selected until each SNA group quota (n = 20) was reached.” |
|  |  | (b) Give reasons for non-participation at each stage |  |
|  |  | (c) Consider use of a flow diagram |  |
| Descriptive data | 14* | (a) Give characteristics of study participants (eg demographic, clinical, social) and information on exposures and potential confounders | **Results - Descriptive**: n=60 (28 M, 32 F); mean age 33.78 (20-60). / No missing data |
|  |  | (b) Indicate number of participants with missing data for each variable of interest |  |
| Outcome data | 15* | Report numbers of outcome events or summary measures | **Results - Outcomes/Tables 1-3**: means and frequencies provided. |
| Main results | 16 | (*a*) Give unadjusted estimates and, if applicable, confounder-adjusted estimates and their precision (eg, 95% confidence interval). Make clear which confounders were adjusted for and why they were included | **Results**: p-values reported for comparisons. |
|  |  | (*b*) Report category boundaries when continuous variables were categorized |  |
|  |  | (*c*) If relevant, consider translating estimates of relative risk into absolute risk for a meaningful time period |  |
| Other analyses | 17 | Report other analyses done—eg analyses of subgroups and interactions, and sensitivity analyses | **Results**: sex and side comparisons; inter- and intra-observer ICCs reported (0.939, 0.987). |
| Discussion | | | |
| Key results | 18 | Summarise key results with reference to study objectives | **Discussion - opening paragraphs & Conclusion**: main associations (GPF–IF distance and molar position differences) summarized. |
| Limitations | 19 | Discuss limitations of the study, taking into account sources of potential bias or imprecision. Discuss both direction and magnitude of any potential bias | **Discussion - Limitations paragraph**: single-center, limited generalizability, no multivariable adjustment, sample size. |
| Interpretation | 20 | Give a cautious overall interpretation of results considering objectives, limitations, multiplicity of analyses, results from similar studies, and other relevant evidence | **Discussion - Interpretation**: clinical implications and call for prospective studies. |
| Generalisability | 21 | Discuss the generalisability (external validity) of the study results | **Discussion - Generalizability**: note regional/ethnic limits; recommend multicenter studies   \|  \| \| --- \| |
| Other information | | | |
| Funding | 22 | Give the source of funding and the role of the funders for the present study and, if applicable, for the original study on which the present article is based | **Declarations - Funding**: “The authors would like to thank the Vice Chancellor for Research, Shiraz University of Medical Science, for supporting this investigation.” |

*Give information separately for exposed and unexposed groups.

**Note:** An Explanation and Elaboration article discusses each checklist item and gives methodological background and published examples of transparent reporting. The STROBE checklist is best used in conjunction with this article (freely available on the Web sites of PLoS Medicine at http://www.plosmedicine.org/, Annals of Internal Medicine at http://www.annals.org/, and Epidemiology at http://www.epidem.com/). Information on the STROBE Initiative is available at www.strobe-statement.org.
